# Aberrant motor contagion of emotions in psychopathy and high-functioning autism

**DOI:** 10.1093/cercor/bhac072

**Published:** 2022-03-24

**Authors:** Lihua Sun, Lasse Lukkarinen, Tuomo Noppari, Sanaz Nazari-Farsani, Vesa Putkinen, Kerttu Seppälä, Matthew Hudson, Pekka Tani, Nina Lindberg, Henry K Karlsson, Jussi Hirvonen, Marja Salomaa, Niina Venetjoki, Hannu Lauerma, Jari Tiihonen, Lauri Nummenmaa

**Affiliations:** Turku PET Centre, University of Turku, Turku FI-20521, Finland; Turku PET Centre, Turku University Hospital, Turku FI-20521, Finland; Department of Nuclear Medicine, Huashan Hospital, Fudan University, Shanghai, 200040, China; Turku PET Centre, University of Turku, Turku FI-20521, Finland; Turku PET Centre, Turku University Hospital, Turku FI-20521, Finland; Psychiatric Hospital for Prisoners, Health Care Services for Prisoners, Turku FI-20251, Finland; Department of Psychiatry, Turku University Hospital, Turku FI-20251, Finland; Department of Psychiatry, Helsinki University Hospital, Helsinki FI-00014, Finland; Turku PET Centre, University of Turku, Turku FI-20521, Finland; Turku PET Centre, University of Turku, Turku FI-20521, Finland; Turku PET Centre, University of Turku, Turku FI-20521, Finland; Department of Medical Physics, Turku University Hospital, Turku, Finland; Turku PET Centre, University of Turku, Turku FI-20521, Finland; Department of Psychiatry, Helsinki University Hospital, Helsinki FI-00014, Finland; Department of Forensic Psychiatry, Helsinki University Hospital, University of Helsinki, Helsinki FI-00014, Finland; Turku PET Centre, University of Turku, Turku FI-20521, Finland; Department of Radiology, University of Turku and Turku University Hospital, Turku FI-20251, Finland; Psychiatric Hospital for Prisoners, Health Care Services for Prisoners, Turku FI-20251, Finland; Psychiatric Hospital for Prisoners, Health Care Services for Prisoners, Turku FI-20251, Finland; Psychiatric Hospital for Prisoners, Health Care Services for Prisoners, Turku FI-20251, Finland; Department of Psychiatry, Turku University Hospital, Turku FI-20251, Finland; Department of Psychiatry, University of Turku, Turku FI-20251, Finland; Department of Clinical Neuroscience, Karolinska Institute, Stockholm SE-11364, Sweden; Center for Psychiatry Research, Stockholm City Council, Stockholm SE-11364, Sweden; Department of Forensic Psychiatry, University of Eastern Finland, Niuvanniemi Hospital, Kuopio FI-70240, Finland; Turku PET Centre, University of Turku, Turku FI-20521, Finland; Turku PET Centre, Turku University Hospital, Turku FI-20521, Finland; Department of Psychology, University of Turku, Turku FI-20251, Finland

**Keywords:** psychopathy, autism, emotion, sociality, motor cortex

## Abstract

Psychopathy and autism are both associated with aberrant social skills and empathy, yet only psychopaths are markedly antisocial and violent. Here, we compared the functional neural alterations underlying these two groups that both have aberrant empathetic abilities but distinct behavioral phenotypes. We studied 19 incarcerated male offenders with high psychopathic traits, 20 males with high-functioning autism, and 19 age-matched healthy controls. All groups underwent functional magnetic resonance imaging while they viewed dynamic happy, angry, and disgusted faces or listened to laughter and crying sounds. Psychopathy was associated with reduced somatomotor responses to almost all expressions, while participants with autism demonstrated less marked and emotion-specific alterations in the somatomotor area. These data suggest that psychopathy and autism involve both common and distinct functional alterations in the brain networks involved in the socioemotional processing. The alterations are more profound in psychopathy, possibly reflecting the more severely disturbed socioemotional brain networks in this population.

## Introduction

The ability to relate with others is a fundamental human skill that is highly automated. Effortless flow of emotional states and goals across individuals facilitates the understanding of intentions and actions and allows us to “tune in” with others (Hatfield et al. 1993; Keysers et al. 2010; Nummenmaa et al. 2012). However, there exists marked variation in the ability to understand others’ needs and goals as well as to take these into account in social interactions. Psychopathy is an extreme case of lacking ability to relate with others despite of the skillful manipulativeness to achieve goals among social interactions (Wilson 1994). It is characterized by recurring antisocial behavior, bold, disinhibited, and egotisti-cal traits, and lacking empathy and remorse (Cooke and Michie 2001). Psychopathy is also causally linked with criminal behavior and violence (Murrie et al. 2004). While the prevalence of psychopathy is around 1% in normal population, it is around 20% in incarcerated offenders (Hare 2003) and 16.4% in Finnish incarcerated offenders (Jüriloo et al. 2014). Because these behavioral and emotional symptoms are persistent and present already in childhood, psychopathy likely has an organic basis.

Neuroimaging studies have found that psychopathic offenders have lower volume in the frontal cortex and in limbic regions, including insula and amygdala (Müller et al. 2008; Tiihonen et al. 2008; Yang et al. 2009; Ermer et al. 2012; Nummenmaa et al. 2021). These structural alterations are accompanied with abnormal responsiveness of the limbic system. Psychopathy is associated with weaker activity in the amygdala and hippocampus, striatum, and cingulate cortices while viewing emotional facial expressions. Psychophysiological and neuroimaging studies have revealed that participants with psychopathic traits show significantly reduced autonomic nervous system responses and frontocortical brain activity toward others’ distress, which is consistent with lowered care motivation (Decety et al. 2013; Meffert et al. 2013). Conversely, stronger responses are observed in the frontal cortical regions (Kiehl et al. 2001; Dolan and Fullam 2009), particularly when viewing violent emotional episodes (Nummenmaa et al. 2021). The distorted limbic outputs combined with dysfunction in executive frontal cortical and social decision-making systems could thus predispose psychopaths to violent and antisocial behavior (Contreras-rodríguez et al. 2014; Contreras-Rodríguez et al. 2015).

Difficulty in relating with other people is a common feature of the autistic and psychopathic phenotype (Marsh 2018). Autism spectrum disorders (ASDs) are also characterized by abnormalities and difficulties in the social domain, and similarly as psychopathy, they have an early onset and neurodevelopmental origin. ASDs are typically manifested as aberrant communication, restricted interests, repetitive behavior, and sensory anomalies (Battle 2013; Lord et al. 2020). ASDs have variable clinical phenotypes from mild to severe, and even wider continuum of social-communicative ability extending into the general population has been proposed (Vecera and Marron 1996; Baron-Cohen et al. 2001). Neuroimaging studies have linked ASD with aberrant structure and function in socioemotional brain networks, such as those involved in the processing of goal-directed actions and biological motion (superior temporal sulcus), theory of mind (medial prefrontal cortex and temporo-parietal junction), and emotion (amygdala) (Harms et al. 2010; Uljarevic and Hamilton 2013; Glerean et al. 2016). Similar to psychopathy, ASD is associated with reduced limbic activation by emotional stimuli, such as happy, fearful, and disgusted faces (Ogai et al. 2003; Kim et al. 2015). It is however controversial whether aberrant socioemotional processing pertains to all emotions versus only a subset of them, and even meta-analyses have provided evidence for both general (Lozier et al. 2014) and emotion-specific effects (Uljarevic and Hamilton 2013).

ASD and psychopathy have a set of common and distinct characteristics. Both ASD and psychopathy are both overrepresented in forensic settings (Im 2016), and aggression is also somewhat common in autistic samples (Kanne and Mazurek 2011). Because early onset childhood conduct disorder is highly predictive of adult psychopathy, it can also be seen as a neurodevelopmental disorder similarly as ASD (Raine 2018). Both ASD and psychopathy are also heritable and may have shared genetic basis (O’Nions et al. 2015; Tiihonen et al. 2020). Despite these shared features, the behavioral phenotypes in psychopathy and ASD also differ in important ways. First, although ASD might be underdiagnosed in forensic settings (Loureiro et al. 2018), the available data show that antisocial behavior is more common in psychopathy than in ASD. Second, while psychopathic individuals can use their superficial charm and glib for manipulating other people (Wilson 1994), autistic individuals have, in general, severe difficulties in maintaining even routine social interactions. Third, the nonsocial domains of ASD (restricted interests, repetitive behavior, and sensory anomalies) are not manifested in psychopathy, which is better characterized by impulsive rather than highly structured behavioral patterns (Cooke and Michie 2001); intellectual disabilities are also not common in psychopaths who tend to be of average intelligence (Hare 2003). Finally, while psychopathy is characterized by impaired mental state attribution for others’ emotions, ASD is associated with impaired cognitive perspective-taking (Jones et al. 2010; Marsh and Cardinale 2012; Lockwood et al. 2013).

Taken together, individuals with both autism and psychopathy can act in ways that implicate lack of empathy toward others, and aberrant functioning in comparable brain systems have been implicated in both conditions. Comparison between autistic and psychopathic individuals’ neural response to socioemotional signals would thus provide a unique opportunity for addressing whether specific perturbations of the socioemotional brain networks are linked with distinct social and antisocial behavioral patterns. However, to our knowledge, no prior study has directly compared functional brain basis of psychopathy and autism.

### The current study

In the current study, we compared neural responses to emotional communicative signals in healthy controls versus incarcerated psychopathic offenders and individuals with ASD. All groups underwent functional magnetic resonance imaging (fMRI) while they viewed dynamic happy, angry, and disgusted facial expressions or listened to laughter and crying sounds. We show that psychopathy is associated with reduced somatomotor responses to almost all expressions, while in ASD, comparable alterations were found only for laughter and disgusted facial expressions. Direct comparison revealed that downregulation of the somatomotor responses to all facial expressions was larger in psychopathy versus ASD.

## Methods

### Subjects

We studied 19 convicted male offenders with high psychopathic traits, 20 males with high-functioning autism, and 19 age-matched healthy controls. Exclusion criteria were psychotic or other severe psychiatric illnesses, autoimmune illnesses, use of opioids, antipsychotic medication other than very small doses for insomnia, current substance abuse, exceptional risk of violence, claustrophobia, and other contraindications for magnetic resonance imaging. The study was approved by the ethical committee of the Hospital District of Southwest Finland and was conducted in accordance of the Helsinki declaration. All participants completed informed consent forms prior to participating.

#### Convicted offenders

Offenders were inmates of the Turku Prison and they had been sentenced for murder (*n* = 5), manslaughter (*n* = 5), attempted manslaughter (*n* = 3), or grievous bodily harm (*n* = 6). Information regarding the study was distributed to the inmates potentially eligible for the study, and volunteers were then evaluated by the prison hospital psychiatrists. Psychiatric diagnoses for offenders were based on prison health care and forensic psychiatric violence risk assessments, forensic psychiatric examination reports concerning legal responsibility, 2 recruitment interviews, and semistructured Psychopathy Checklist-revised (PCL-R) interviews. Final consensus diagnoses were made by two medical specialists (M.S. and H.L.), both with 13–25 years of experience in the field of prison psychiatry, which was also assisted by a psychologist (N.V.) with a 15-year working history in the Psychiatric Hospital for Prisoners. Offenders were escorted by 2 prison guards to the local research institute for the brain imaging study. More detailed clinical information of offenders is found in Supplementary Table S1.

None of the offender group was psychotic nor suffered from a significant mood disorder, as assessed via a SCID-I interview (Spitzer et al. 1992). The group consisted of 16 participants with antisocial personality disorders, as defined by DSM-5 criteria (Battle 2013), and 3 who did not fulfill the criterion of conduct disorder before the age of 15 years but only the other criteria of antisocial personality. History of excessive alcohol use was present in 13 participants, and 18 participants had self-reported or documented use of illegal substances, including black market benzodiazepines, pregabalin or opioids, cannabis, amphetamines, gamma-hydroxybutyrate, MDPV, anabolic steroids, and cocaine. Information concerning the severity of abuse was considered unreliable.

Psychopathy scores of the offenders were evaluated with semistructured interview by experienced forensic psychiatrists or psychologists based on the PCL-R (Hare 2003); Levenson Self-Report Psychopathy Scale (LSRP) questionnaires (Levenson et al. 1995) for offenders were also documented as untrusted data. Psychopathy scores of healthy controls and participants with ASD were based on the LSRP questionnaire. LSRP measures 2 dimensions of psychopathy, with the primary psychopathy score indicating inclination to lie, lack of remorse, and callousness and the secondary psychopathy score indicating impulsivity, short temper, and low toleration for frustration.

#### Participants with ASD

Participants in the ASD group were volunteers from the Helsinki and Turku University Hospital Neuropsychiatric Clinic, and 1 participant was also recruited from the Neuropsychiatric Clinic Proneuron in Espoo. Based on patient history, accessible information from births records, well-baby clinics, and school health care, the ASD diagnoses were verified by research psychologist, neurologist, and psychiatrist following the DSM-5 criteria. An additional current Autism Diagnostic Observation Schedule (ADOS) assessment (Lord et al. 2012) was also used to clarify the ASD diagnostics. All ASD participants were diagnosed with ASD, 6 also with ADHD and 8 with other mood and anxiety disorders. Healthy participants and ASD group also completed the autism-spectrum quotient (AQ) questionnaire (Bishop et al. 2004). None of the ASD participants had currently severe mental disorder, as assessed via the SCID-I interview. Dopaminergic medications (antipsychotics, psychostimulants, and bupropion) were withdrawn before measurements, but 4 participants who had SSRI medication could not be withdrawn. Clinical information of the ASD participants is found in Supplementary Table S2.

#### Healthy controls

The control participants were screened for medical conditions from their patient histories, and their use of prescribed medication was double-checked from the Finnish medical database. Clinical information of the control participants is found in Supplementary Table S3.

### Facial expression task

**Fig. 1:**
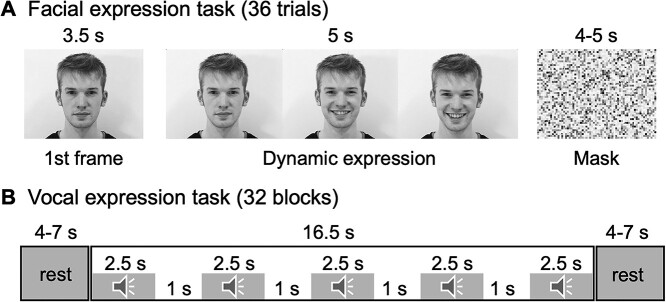
Experimental design for the facial expression task A) and vocal expression task B). Color images and videos were used in the facial expression task.

In the emotional facial expression task (Fig. 1A), participants viewed short video clips (5 s) of dynamic facial expressions of joy, disgust, and anger selected from ADFES database (van der Schalk et al. 2011). All clips begun with a neutral face, which was followed by a dynamic display of the facial expression. Prior to each clip, participants were shown the first frame of the video (i.e. neutral face) for 3.5 s to avoid peaks in low-level visual activation due to simultaneous visual stimulus and motion onset. This was followed by the dynamic expression from neutral to full expression, with the full-blown phase held until the end of the clip. Each stimulus was followed by a random 4–8 s of rest period. Again, to avoid peaks in low-level visual cortical activations, a scrambled picture of the upcoming model was shown during the rest period. To keep participants focused on the task, 4 trials (out of 36 trials in total) contained a still picture of the neutral face instead of the video clip. Participants were asked to press the response button as soon as they detected a trial without any facial motion. These trials were excluded from the analysis. Previous work shows that these dynamic stimuli elicit consistent and expression-specific neural responses in emotion and face perception circuits (Volynets et al. 2020).

### Vocal expression task

In the vocal expression task (Fig. 1B), the participants listened to short laughter and crying vocalizations and control stimuli which were generated by time-domain scrambling of the original sounds. The original stimuli have been validated and described in detail in O’Nions et al. (2017). The experiment was run using a blocked design. In each 16.5-s block, 5 2.5-s stimuli from 1 category (i.e. laughter, crying sounds, scrambled laughter, or scrambled crying sounds) were played with a 1-s silent period between stimuli. Order of the blocks were randomized. The blocks were interspersed with rest blocks lasting for 4–7 s. To keep participants focused on the task, an animal sound (vocalization of an alpaca) was presented randomly during 50% of the rest blocks. The participants were instructed to press the response button whenever they heard the alpaca, and the behavioral outcomes were inspected for the focus of attention. A total of 32 blocks (8 blocks per stimulus type) were run.

### fMRI acquisition and preprocessing

The magnetic resonance imaging (MRI) data were acquired with Phillips Ingenuity TF PET/MR 3T whole-body scanner. High-resolution (1 mm^3^) structural brain images were acquired using a T1-weighted (T1w) sequence (time repetition [TR] = 9.8 ms, time echo [TE] = 4.6 ms, flip angle = 7°, 250 mm FOV, 256 × 256 reconstruction matrix). Radiologist screened the images for structural abnormalities. Functional data were acquired using a T2*-weighted echo-planar imaging sequence (TR = 2600 ms, TE = 30 ms, 75° flip angle, 240 mm FOV, 80 × 80 reconstruction matrix, 62.5 kHz bandwidth, 3.0 mm slice thickness, 45 interleaved slices acquired in ascending order without gaps). A total of 206 (facial expression task) or 290 (laughter task) functional volumes were acquired. We used fMRIPrep 1.3.0.2 to preprocess the MRI data (Esteban et al. 2019). Anatomical T1w reference images were processed following steps: correction for intensity nonuniformity, skull-stripping, brain surface reconstruction, spatial normalization to the ICBM 152 Nonlinear Asymmetrical template version 2009c (Fonov et al. 2009) using nonlinear registration with antsRegistration (ANTs 2.2.0), and brain tissue segmentation. fMRI data were processed following steps: coregistration to the T1 reference image, slice-time correction, spatial smoothing with a 6-mm Gaussian kernel, automatic removal of motion artifacts using ICA-AROMA (Pruim et al. 2015), and resampling to the MNI152NLin2009cAsym standard space. Quality of images was assessed via the visual reports of fMRIPrep and was inspected manually in accord to the whole-brain field of view coverage, proper alignment to the anatomical images, and signal artifacts. All functional data were retained in the analysis. Quality of images was visually checked and also inspected based on fmriprep’s visual reports. No images had >25% of frames with >1-mm frame displacement (Parkes et al. 2018).

### Full-volume GLM data analysis

The fMRI data were analyzed in SPM12 (Wellcome Trust Center for Imaging, London, UK, (http://www.fil.ion.ucl.ac.uk/spm). The whole-brain random effects model was applied using a 2-stage process with separate first and second levels. For each participant, GLM was used to predict the regional effects of task parameters on blood oxygen level–dependent (BOLD) indices of activation. In the facial expression task, contrast images were generated for dynamic happy, angry, or disgusted facial expressions versus static neutral faces (i.e. the initial 3.5 s of each video without motion) and were subjected to second-level analyses for population-level inference. In the vocal expression task, contrast images were generated for laughter or crying sound versus corresponding scrambled sounds and were subjected to second-level analyses. We first tested the task-dependent activations in each group and conducted the between-group comparisons for each effect of interest. In addition to between-group comparisons, in a control analysis, we also fitted a multiple regression model where the primary LSRP scores and AQ scores were used as regressors. By controlling the effect of the other, this provided additional information regarding the specific effect of these factors across all the subject groups. The secondary LSRP scores were significantly correlated with the AQ scores when the groups were pooled (*r* = 0.37, *P* = 0.005) and therefore were not applied in the control analysis. Statistical threshold was set at *P* < 0.05, FDR-corrected at cluster level.

### Region of interest analysis

To visualize the between-group differences, BOLD signals in anatomically defined regions of interest (ROIs) were also analyzed. ROIs were selected considering their important roles in socioemotional processing. These ROIs included anterior, middle, and posterior cingulate cortices (CCs), precuneus, amygdala, caudate, putamen, and insula defined by the AAL atlas (Tzourio-Mazoyer et al. 2002). We also included the subregions of motor area, which are parceled in the Juelich Atlas with masks generated using the SPM Anatomy toolbox (Eickhoff et al. 2005). These subregions include the primary motor cortex (M1) corresponding to Brodmann areas (BAs) 4a and 4b; the supplementary motor area (M2) corresponding to BA6 (Geyer 2004); the primary somatosensory cortex (S1), including BA3a, BA3b, BA1, and BA2 (Geyer et al. 2000; Grefkes et al. 2001); and the secondary somatosensory cortex (S2), including parietal operculum 1-4 (Eickhoff et al. 2006). Regional beta weights were estimated from first-level contrast images of each participant using the MarsBaR toolbox (Brett et al. 2002). ROI data were analyzed using 2-sample *t*-test in R statistical software (version 3.6.3).

## Results

### Psychopathy and autism evaluation in the studied groups

Basic information of participants is summarized in Table 1.

**Table 1:** Basic characteristics of the participants.

Groups	Control	ASD	Psychopathy
Age	28.53 (7.69)	27.85 (5.56)	31.16 (6.49)
Education level			
Interrupted primary school	0	0	2
Primary school	0	3	12
Second degree	10	14	5
University degree	9	3	0
Psychopathy			
PCL-R	—	—	26.47 (6.24)
LSRP primary psychopathy	21.95 (3.05)	23.30 (3.95)	30.67 (5.96)^a^
LSRP secondary psychopathy	13.47 (2.97)	16.60 (3.28)	19.8 (3.14)^a^
Autism			
AQ	10.95 (3.44)	27.65 (5.64)	19.63 (6.43)^a^
ADOS	—	11.30 (4.34)	—

Note: — indicates data not available.

^a^Indicates data not trusted.

In the psychopathy group, interview based PCL-R measures were conducted since their self-reported data were considered unreliable due to the nature of psychopathy. This was also supported by a lack of significant correlation for PCL-R scores with either primary LSRP scores (*r* = 0.3, *P* = 0.3) or secondary LSRP scores (*r* = 0.23, *P* = 0.4). However, both primary and secondary LSRP scores of the psychopathy group were significantly higher than the other groups (Supplementary Fig. S1).

In the ASD group, AQ scores were significantly higher compared to controls (*t* = 11.14; *P* < 0.001). While there was no statistical difference between ASD group and controls for LSRP primary psychopathy scores (*t* = 1.20; *P* = 0.24), the ASD group had higher secondary psychopathy score (*t* = 3.12; *P* = 0.003).

### Regional responses to positive emotional stimuli

**Fig. 2:**
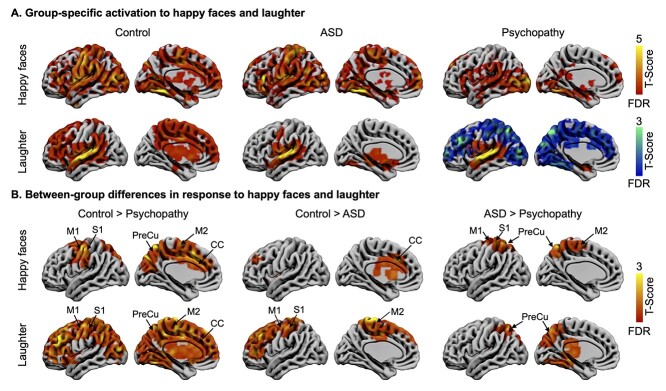
Brain responses to happy faces and social laughter. A) Responses to happy faces and laughter separately for each group. Hot color indicates activation and cool color indicates deactivation. B) Between-group differences in response to happy faces and laughter. Data are thresholded at *P* < 0.05 with FDR cluster-level correction. S1 = primary somatosensory cortex, S2 = secondary somatosensory cortex, M1 = primary motor cortex, M2 = supplementary motor area, and PreCu = precuneus.

In the control group, happy faces elicited activation in the occipital cortex; fusiform gyrus; CC; motor area, including the primary (S1) and secondary (S2) somatosensory cortex and primary (M1) and supplementary motor (M2) areas; medial frontal cortex (MFC); middle temporal gyrus (MTG) and superior temporal gyrus (STG); precuneus; cuneus; amygdala; hippocampus; striatum; and thalamus (Fig. 2A). Social laughter sounds elicited activation in the primary and secondary auditory cortices, CC, motor area, MFC, MTG, and STG, precuneus, amygdala, hippocampus, striatum, and thalamus. These activations by both happy faces and laughter were weakened in the autistic individuals and were markedly abolished in the psychopathy group, with the exception of the temporal activations (Fig. 2A). In the psychopathy group, large-scale deactivation was also observed for laughter.

This was confirmed in direct between-group contrasts (Fig. 2B). Compared to controls, the psychopathy group showed dampened responses to happy faces and laughter in motor area, CC, and precuneus. Dampened activation in response to laughter expanded largely to frontal and posterior brain areas and subcortical regions. Compared to controls, ASD group showed dampened responses in the middle and anterior CCs to happy faces and in the motor area (also expanding frontally) to laughter. Compared to ASD group, psychopathy group showed dampened response in motor area to happy faces and in precuneus to laughter.

### Regional responses to negative emotional stimuli

**Fig. 3:**
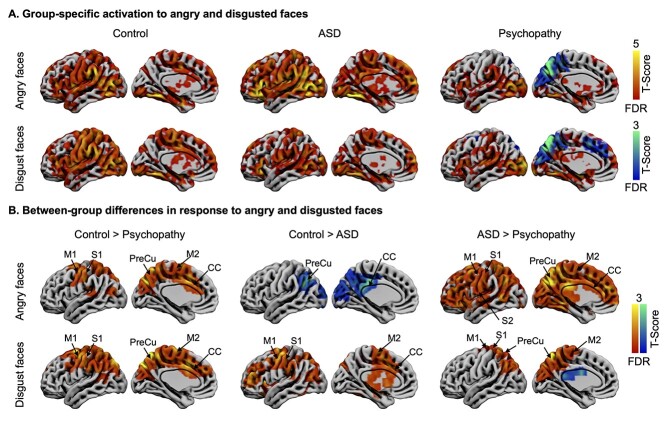
Brain responses to angry and disgusted faces. A) Responses to angry and disgusted faces separately for each group. B) Between-group differences in responses to angry and disgusted faces. Data are thresholded at *P* < 0.05 with FDR cluster-level correction.

In controls, both angry and disgusted faces elicited activation in the occipital cortex, FFA, CC, the motor area, MFC, MTG and STG, precuneus, amygdala, hippocampus, striatum, and thalamus (Fig. 3A). Comparable activation of these regions was found in autistic individuals, while similarly as for happy faces and laughter, activities in these brain regions in psychopathic individuals were markedly abolished (Fig. 3A).

This was also confirmed in direct between-group contrast (Fig. 3B). Compared to controls, participants with psychopathy showed dampened activation in CC, motor area, and precuneus to both angry and disgusted faces. Compared to controls, ASD group also showed dampened activation in the CC, motor area, and precuneus to disgusted faces. However, in response to angry faces, ASD group showed increased activation in precuneus and posterior CCs. Compared to ASD group, the psychopathy group showed global deactivation to angry faces and damped activation in motor area and precuneus to disgusted faces.

Crying sound elicited activation mainly in the primary and secondary auditory cortices and in nearby regions (Supplementary Fig. S2). However, group comparisons did not show statistical differences. We also investigated whether the self-reported LSRP and AQ scores, while controlling for each other, were specifically associated with brain responses to the facial and vocal expression stimuli (Supplementary Fig. S3). In line with the group-level findings, data showed that LSRP primary score was specifically associated with reduced response to laughter in the CC, thalamus lateral prefrontal cortex, somatomotor area, and precuneus. Also, AQ score was specifically associated with increased brain response to angry faces in CC, somatomotor area, and precuneus.

### ROI analysis

**Fig. 4:**
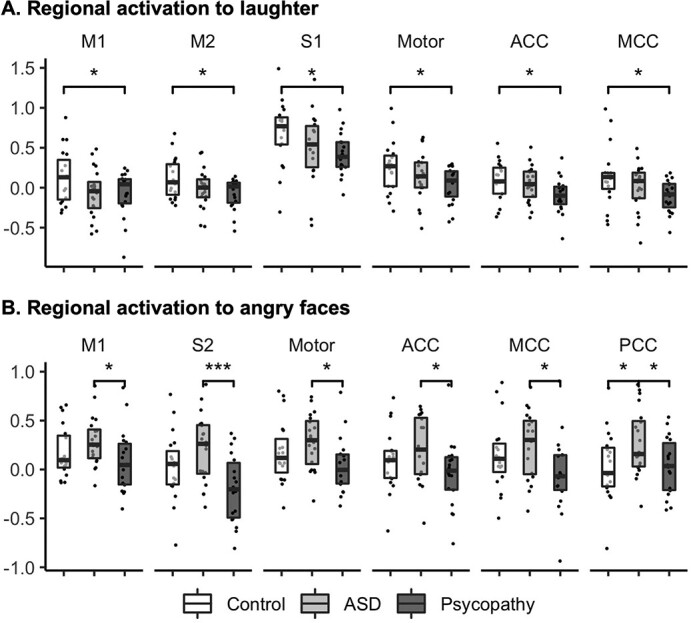
Region-of interest analysis for laughter (A) and angry faces (B). Between group comparisons were conducted using student’s *t*-test, with significance levels marked: ^*^P < 0.05, ^***^P < 0.01. motor = combined region of M1, M2, S1, and S2; data involved both hemispheres.

ROI analysis demonstrated between-group differences that were in accord with the full-volume analysis (Fig. 4). In response to laughter (Fig. 4A), psychopathy group showed reduced activation in M1, M2, S1, and the combined somatomotor areas (M1, M2, S1, and S2 together), anterior cingulate cortex (ACC), and middle cingulate cortex (MCC) compared to controls. There were no statistically significant differences between controls and ASD group, although numerically mean activity was strongest in controls and weakest in psychopathy group in most ROIs.

In response to angry faces (Fig. 4B), psychopathy group demonstrated reduced activation in ROIs, including the M1, S2, whole motor area, ACC, MCC, and posterior cingulate cortex (PCC) compared to ASD group. ASD group also showed increased activation in the PCC compared to controls. No between-group differences were found for crying sounds and happy faces. For disgusted faces, psychopathy group showed reduced activation in MCC compared to controls (data not shown). Subcortical BOLD activity in the amygdala, insula, and striatum did not show between-group differences in the tasks.

## Discussion

Incarcerated offenders with psychopathic traits and patients with high-functioning autism showed both common and unique alterations in the brain responses to positive and negative facial and vocal social communicative signals. Compared with controls, offenders showed lowered brain activation toward all communicative signals except for crying sounds. Weaker activity was observed in somatosensory, motor, and CC. This effect was less pronounced in the patients with ASD and was observed primarily for laughter and disgusted facial expressions. Direct comparison between psychopathic offenders and patients with ASD revealed that the somatomotor responses were weaker in offenders. Altogether, our data show that alterations in somatomotor processing of emotional signals is a common characteristic of criminal psychopathy and autism, yet the degree and specificity of these alterations distinguishes between these two groups. The higher overall degree of alterations in the psychopathic offenders might explain this phenotype manifested by both lacking the ability to relate with others as well as violent behavior.

Our main finding was that somatomotor “mirroring” of vocal and facial emotional expressions was altered in both criminal offenders and participants with ASD and that the somatosensory and motor responses to emotional signals were more reduced in the criminal offenders than in the ASD group. This accords with previous studies that have found reduced brain activation during passive observation of others’ distress (Meffert et al. 2013) or affective memory tasks (Kiehl et al. 2001) in participants with psychopathic traits. Psychopathic offenders also show less behavioral contagion of laughing and yawning (Hagenmuller et al. 2012), and recent structural imaging study demonstrated that both criminal psychopathy as well as psychopathy-like traits in healthy controls are associated with lower volume in the somatosensory cortices (Nummenmaa et al. 2021).

Seeing others in a particular emotional state often triggers automatically the corresponding behavioral and somatic representation of that emotional state in the observer (Dimberg and Thunberg 1998; Wild et al. 2001). Neuroimaging studies have confirmed that such somatomotor contagion of emotions is subserved by common neural activation for the perception and experience of states, such as pain (Singer et al. 2004; Jackson et al. 2005; Saarela et al. 2007), disgust (Wicker et al. 2003), and pleasure (Jabbi et al. 2007), allowing to “tune in” or “sync” with other individuals (Keysers et al. 2010; Nummenmaa et al. 2012). Furthermore, damage to somatosensory cortex (Adolphs et al. 2000) and their inactivation by transcranial magnetic stimulation (Pourtois et al. 2004) also impair recognition of emotions from facial expressions. The widespread aberrant responsivity of the somatosensory cortex in psychopathic offenders may explicate their asocial character, lack of empathy, and egotistical traits (Cooke and Michie 2001). As mirroring of others’ emotions and particularly distress plays a crucial role in empathy and inhibition of violent behavior (Blair 2001; Karjalainen et al. 2017), impaired somatic and motor contagion of others’ emotions may render psychopaths susceptible to antisocial behavior and violence.

The participants with ASD also had lowered somatomotor responses to emotional signals, although this effect was less profound than in the offenders. Prior work shows that autistic individuals have difficulties in recognizing specific emotions (Clark et al. 2008; Harms et al. 2010; Uljarevic and Hamilton 2013) as well as difficulties in automatic mimicking facial expressions (McIntosh et al. 2006; Oberman et al. 2009). Some studies have also shown that patients with ASD have deficient motor intention understanding ability, which is possibly linked with aberrant motor cognition (Cattaneo et al. 2007; Boria et al. 2009; Casartelli et al. 2016). In line with these studies, functional neuroimaging experiments show that high-functioning autism hinders the brain from synchronizing with those of others while viewing naturalistic social interaction, which is indicative of aberrant automatic tuning in with others’ mental states (Hasson et al. 2009; Salmi et al. 2013). The present data highlight how the aberrant activity of the somatosensory and motor cortices may also contribute to these impairments.

Although both psychopathy and ASD groups showed, in general, reduced responses to the vocal and facial emotional expressions, the specific patterns of these alterations differed across the groups. Overall, the emotional expressions evoked weaker responses in the psychopathic than autistic individuals, and for all facial expressions, this effect was observed in the primary somatosensory, primary, and supplementary motor cortices. Because motor responses to social communicative signals are fundamental for establishing social bonds between individuals (Iacoboni et al. 2005; Keysers and Gazzola 2007) and are important for the formation of empathic responses (Gallese 2001; Leslie et al. 2004; Warren et al. 2006), the present observed aberrant motor contagion may reflect the shared component of the socioemotional deficits in autism and psychopathy. Laughter expressions elicited to large-scale deactivation outside the auditory cortices only in offenders. Laugher is a universally recognized prosocial signal that is used for bonding purposes, rather than an expression of positive emotional state (Scott et al. 2014), and many of the characteristics defining psychopathy are related to abnormal socioemotional interaction. It is thus possible that the aberrant neural responses to bonding signals, such as laughter, could link with the antisocial traits in psychopathy. Additionally, for angry faces, the difference between psychopathy and ASD groups was markedly widespread, with psychopathy group showing significantly reduced responses across the medial and lateral frontal cortices in comparison with the autistic patients. These data suggest that autism-associated hypersensitivity of the neural systems responding to anger and hyposensitivity to prosocial cues, such as laugher, may explain the distinct patterns of social interaction and communication deficits in psychopathy and autism.

The current study also bears limitations. Although we aimed at recruiting prisoner volunteers not using antipsychotics, antidepressants, or anxiolytics, it was not possible to recruit a completely drug-naive sample. The convicted offenders and healthy controls and participants with ASD also differ from each other regarding the available quality and quantity of social interaction, leisure time activities, education levels, and so forth. Ideally, this kind of study should thus also involve a forensic but nonpsychopathic sample. Despite of these mentioned limitations, however, our reported between-group differences were supported by the control analysis based on cautiously trusted common measures. Our data are cross-sectional in nature and cannot resolve the potential causal link between the functional alterations and psychopathy and autism. Further, because our focus was on criminal psychopathy, we decided against completing the laborious and time-consuming PCL-R protocol for the healthy and nonincarcerated sample. We only included male participants in the current study and findings may not generalize to females.

In summary, our findings suggest that aberrant neural activity in somatomotor areas may be a common mechanism underlying the asocial behavior in psychopathy and autism, while its severity and selectivity in response to different types of social communicative signals set these disorders apart. These data suggest that distinct conditions associated with social information processing abnormalities might share common neurobiological substrates despite distinct behavioral and clinical phenotypes.

## Supplementary Material

Supplementary_Figure_S1_bhac072

Supplementary_Figure_S2_bhac072

Supplementary_Figure_S3_bhac072

Supplementary_Table_S1_bhac072

Supplementary_Table_S2_bhac072

Supplementary_Table_S3_bhac072
