# Supplementary material for: Aberrant motor contagion of emotions in psychopathy and high-functioning autism
Source: Cereb Cortex. 2022 Mar 24;33(2):374–84. doi: 10.1093/cercor/bhac072 (PMC9837606; doi:10.1093/cercor/bhac072)
Supplement: Supplementary_Figure_S1_bhac072 [file supplementary_figure_s1_bhac072.docx]

**Supplementary Figure S1.** Between-group comparisons of LSRP primary psychopathy (**A**), LSRP secondary psychopathy (**B**), and Autism-Spectrum Quotient (AQ) scores (**C**). ** p < 0.01, *** p < 0.001, **** p < 0.0001.
