# Supplementary material for: Aberrant motor contagion of emotions in psychopathy and high-functioning autism
Source: Cereb Cortex. 2022 Mar 24;33(2):374–84. doi: 10.1093/cercor/bhac072 (PMC9837606; doi:10.1093/cercor/bhac072)
Supplement: Supplementary_Figure_S2_bhac072 [file supplementary_figure_s2_bhac072.docx]

**
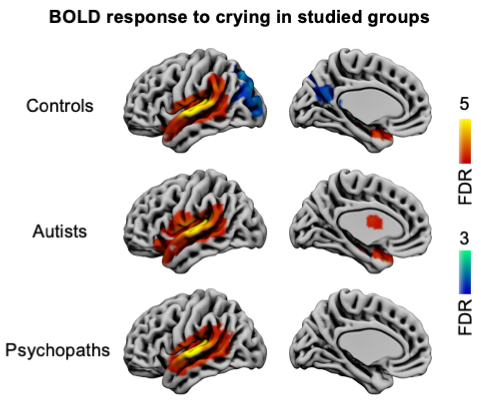
**

**Supplementary Figure S2.** Brain responses to crying sound in the studied groups. There were no between-group differences. Results are thresholded at p < 0.05 with FDR cluster-level correction; left hemispheres were presented for visualization.
