# Supplementary material for: Aberrant motor contagion of emotions in psychopathy and high-functioning autism
Source: Cereb Cortex. 2022 Mar 24;33(2):374–84. doi: 10.1093/cercor/bhac072 (PMC9837606; doi:10.1093/cercor/bhac072)
Supplement: Supplementary_Figure_S3_bhac072 [file supplementary_figure_s3_bhac072.docx]

**Supplementary Figure S3**. LSRP primary score and AQ-score related brain responses to the socio-emotional stimuli when combining all groups. **A.** LSRP primary score related brain activity while the AQ score was controlled. **B.** AQ score related brain activity while the LSRP score was controlled. Data are thresholded at p < 0.05 with FDR cluster-level correction; left hemispheres were presented for visualization.
