# Supplementary material for: Aberrant motor contagion of emotions in psychopathy and high-functioning autism
Source: Cereb Cortex. 2022 Mar 24;33(2):374–84. doi: 10.1093/cercor/bhac072 (PMC9837606; doi:10.1093/cercor/bhac072)
Supplement: Supplementary_Table_S1_bhac072 [file supplementary_table_s1_bhac072.docx]

**Table S1.** Clinical and sociodemographic characteristics of the convicted offenders.

| **Patient** | **Age** | **Diagnosis** | **Medication** | **LSRP Primary Score** | **LSRP Secondary Score** | **AQ Score** | **PCL-R Score** |  |
| --- | --- | --- | --- | --- | --- | --- | --- | --- |
| 1 | 27 | AA, BP, HC, SA | Hydroxyzine, Fluoxetine,  Quetiapine (stopped 2 days before) | 26 | 21 | 17 | 28 |  |
| 2 | 31 | AA, ADHD, APD, HCV, SA | Levothyroxine, Amitriptyline | - | - | 12 | 29 |  |
| 3 | 24 | AA, APD, HCV, PD, SA | Citalopram, Buspirone, Propranolol | 28 | 15 | 11 | 36 |  |
| 4 | 31 | AA, ADHD, APD, SA | Hydroxyzine, Melatonin | 34 | 24 | 25 | 28 |  |
| 5 | 25 | APD, SA | Melatonin, Hydroxyzine,  Escitalopram*, Quetiapine*, Levomepromazine* (*stopped 3 days before) | 37 | 21 | 21 | 29 |  |
| 6 | 45 | None | None | 21 | 15 | 14 | 20 |  |
| 7 | 29 | AA, APD, SA | None | 34 | 18 | 16 | 27 |  |
| 8 | 39 | APD, MAD, SA | Hydroxyzine | 32 | 18 | 13 | 33 |  |
| 9 | 26 | ADHD, APD, HCV, SA | Hydroxyzine | 31 | 23 | 25 | 33 |  |
| 10 | 25 | APD, MAD, SA | Mirtazapine (stopped 1 day before) | 19 | 15 | 35 | 21 |  |
| 11 | 30 | AA, APD, BP, HCV, SA | Melatonin,  Buspirone*, Quetiapine* (*stopped 11 days before) | 36 | 21 | 14 | 23 |  |
| 12 | 23 | AA, APD, BP, SA | Quetiapine, Propranolol, Amitriptyline, Hydroxyzine, | 25 | 19 | 25 | - |  |
| 13 | 25 | AA, SA | Quetiapine, Amitriptyline | - | - | - | 20 |  |
| 14 | 28 | SA | None | 31 | 21 | 21 | 20 |  |
| 15 | 41 | AA, ADHD, APD, BP, MAD, SA | Hydroxyzine, Buspirone, Risperidone, Atomoxetine | - | - | - | - |  |
| 16 | 35 | AA, APD, SA | Melatonin, Hydroxyzine, Propranolol,  Quetiapine (stopped 1 day before) | 41 | 21 | 24 | 30 |  |
| 17 | 40 | AA, APD, HCV, SA | Melatonin | - | - | - | 24 |  |
| 18 | 32 | AA, APD, SA | Hydroxyzine, Cetirizine, Melatonin, Amitriptyline, Mirtazapine, Venlafaxine | 32 | 20 | 18 | 16 |  |
| 19 | 36 | AA, ADHD, APD, HCV, SA | None | 33 | 25 | 23 | 35 |  |

APD = antisocial personality disorder, ADHD = Attention-deficit hyperactivity disorder, MAD = Mood and Anxiety Disorder, AA = Alcohol abuse, PD = panic disorder, SA = Substance abuse, BP = Bordeline personality, HCV = Hepatitis C.
