# Supplementary material for: Aberrant motor contagion of emotions in psychopathy and high-functioning autism
Source: Cereb Cortex. 2022 Mar 24;33(2):374–84. doi: 10.1093/cercor/bhac072 (PMC9837606; doi:10.1093/cercor/bhac072)
Supplement: Supplementary_Table_S2_bhac072 [file supplementary_table_s2_bhac072.docx]

**Table S2.** Clinical and sociodemographic characteristics of the ASD patients.

| **Patient** | **Age** | **Diagnosis** | **Medication** | **LSRP Primary Score** | **LSRP Secondary Score** | **AQ Score** | **ADOS Score** |
| --- | --- | --- | --- | --- | --- | --- | --- |
| 1 | 27 | ASD, MAD | None | 24 | 18 | 37 | 16 |
| 2 | 29 | ASD | None | 26 | 16 | 27 | 7 |
| 3 | 25 | ASD | None | 18 | 14 | 31 | 10 |
| 4 | 20 | ASD, MAD | None | 29 | 14 | 30 | 18 |
| 5 | 24 | ASD, ADHD | Melatonin | 19 | 15 | 35 | 7 |
| 6 | 29 | ASD, MAD | Fluoxetine | 29 | 15 | 25 | 15 |
| 7 | 34 | ASD | None | 22 | 19 | 28 | 7 |
| 8 | 40 | ASD, ADHD | Zolpidem (stopped 1 day before) | 30 | 14 | 29 | 16 |
| 9 | 23 | ASD, ADHD | None | 31 | 21 | 27 | 9 |
| 10 | 33 | ASD, MAD | Levothyroxine, Cetirizine, Escitalopram | 22 | 14 | 32 | 7 |
| 11 | 26 | ASD, MAD | Melatonin | 22 | 16 | 19 | 12 |
| 12 | 31 | ASD | None | 25 | 23 | 32 | 13 |
| 13 | 25 | ASD, ADHD | None | 20 | 19 | 27 | 14 |
| 14 | 38 | ASD, MAD | Venlafaxine | 24 | 21 | 22 | 2 |
| 15 | 25 | ASD | None | 20 | 12 | 33 | 12 |
| 16 | 21 | ASD | None | 22 | 10 | 18 | 6 |
| 17 | 26 | ASD, MAD | Escitalopram | 19 | 17 | 26 | 17 |
| 18 | 29 | ASD, MAD | Vortioxetine, Bupropion (stopped 5 days before) | 20 | 18 | 24 | 14 |
| 19 | 20 | ASD, ADHD | Melatonin | 24 | 20 | 34 | 14 |
| 20 | 20 | ASD, ADHD | None | 20 | 16 | 17 | 14 |

Note: ASD= Asperger´s syndrome, ADHD=Attention-Deficit/Hyperactivity disorder, MAD=Mood and Anxiety Disorder
