# Supplementary material for: Aberrant motor contagion of emotions in psychopathy and high-functioning autism
Source: Cereb Cortex. 2022 Mar 24;33(2):374–84. doi: 10.1093/cercor/bhac072 (PMC9837606; doi:10.1093/cercor/bhac072)
Supplement: Supplementary_Table_S3_bhac072 [file supplementary_table_s3_bhac072.docx]

**Table S3.** Sociodemographic characteristics of the healthy controls.

| **Patient** | **Age** | **LSRP Primary Score** | **LSRP Secondary Score** | **AQ Score** |  |
| --- | --- | --- | --- | --- | --- |
| 1 | 30 | 24 | 10 | 9 |  |
| 2 | 21 | 28 | 13 | 10 |  |
| 3 | 22 | 24 | 18 | 18 |  |
| 4 | 23 | 24 | 20 | 15 |  |
| 5 | 21 | 26 | 12 | 16 |  |
| 6 | 34 | 20 | 10 | 12 |  |
| 7 | 47 | 18 | 10 | 12 |  |
| 8 | 23 | 19 | 15 | 9 |  |
| 9 | 31 | 19 | 12 | 11 |  |
| 10 | 20 | 20 | 15 | 14 |  |
| 11 | 43 | 24 | 14 | 4 |  |
| 12 | 25 | 17 | 12 | 9 |  |
| 13 | 26 | 26 | 13 | 6 |  |
| 14 | 28 | 20 | 11 | 13 |  |
| 15 | 32 | 22 | 18 | 7 |  |
| 16 | 37 | 20 | 12 | 11 |  |
| 17 | 33 | 20 | 13 | 9 |  |
| 18 | 24 | 22 | 11 | 9 |  |
| 19 | 22 | 24 | 17 | 14 |  |
